# Central Nervous System Causes of Sudden Unexpected Death: A Comprehensive Review

**DOI:** 10.7759/cureus.20944

**Published:** 2022-01-04

**Authors:** Azizah S Alotaibi, Rabya A Mahroos, Samia S Al Yateem, Ritesh G Menezes

**Affiliations:** 1 College of Medicine, King Fahd Hospital of the University, Imam Abdulrahman Bin Faisal University, Dammam, SAU; 2 Department of Pathology, College of Medicine, King Fahd Hospital of the University, Imam Abdulrahman Bin Faisal University, Dammam, SAU

**Keywords:** molecular autopsy, forensic autopsy, cns-related pathology, central nervous system, sudden unexpected death

## Abstract

Sudden unexpected death due to central nervous system (CNS)-related pathologies though far less common than cardiac causes still account for a substantial proportion of sudden deaths that occur worldwide. This review covers the most common causes of sudden unexpected death due to CNS-related pathologies encountered by forensic pathologists. These include intracerebral hemorrhage, aneurysmal subarachnoid hemorrhage, ischemic stroke, epilepsy, brain tumors, and infectious causes. Related rare causes are not discussed and are beyond the scope of this review. The role of neuroimaging and genetic testing as autopsy ancillary investigations in such sudden deaths is also discussed.

## Introduction and background

Sudden natural death is one of the intriguing cases seen in forensic pathology. It is defined as natural unexpected nontraumatic death in apparently healthy individuals occurring instantaneously or in less than 24 hours after the onset of clinical symptoms [1]. It is most often the result of cardiovascular disease, which was reported to be the cause of sudden death in 56.4%-73% of cases, 58% of which was caused by coronary artery disease [2-3]. The most common non-cardiac causes of sudden death were epilepsy (23.8%), intracerebral hemorrhage (23.8%), asthma (16.1%), and pulmonary embolism (12.5%), and the cause of death was undetermined in 4.3% [3]. Sudden death due to central nervous system (CNS) causes accounted for 15% of all cases of noncardiac deaths and was one of the most common non-cardiac causes of sudden death [4].

Some of the notable intracranial causes of sudden unexpected death include intracranial hemorrhage, ischemic stroke, epilepsy, brain tumors, meningitis, and cerebral abscess (Table 1) [5]. The majority of sudden natural deaths occur outside hospitals and because neuropathology autopsies are uncommonly performed, a cardiac cause of death is most often assumed and neurologic causes could be easily missed [4]. A study examined the numbers of sudden neurologic death in apparent sudden cardiac death cases [4]. Of 335 cases, 18 (5.4%) had an acute neurologic cause of death [4]. Identifying cases of sudden neurologic death carries great significance, as it would provide a better estimation in epidemiological studies in terms of disease burden and mortality rates.

**Table 1:** Notable CNS causes of sudden unexpected death

CNS causes of sudden unexpected death
Intracerebral hemorrhage
Aneurysmal subarachnoid hemorrhage
Ischemic stroke
Epilepsy
Brain tumors
Infectious causes

This review covers the most common causes of sudden unexpected death due to CNS-related pathologies encountered by forensic pathologists. Related rare causes are not discussed and are beyond the scope of this review. The role of neuroimaging and genetic testing as autopsy ancillary investigations in such sudden deaths is also discussed.

## Review

CNS-related causes of sudden unexpected death

Vascular Malformations 

Vascular malformations constitute one of the causes of intracerebral hemorrhage among others including brain neoplasms, coagulopathies, and cerebral and venous sinus thrombosis [6]. Arteriovenous malformations (AVMs) of the brain are abnormal vascular anomalies in which a connection exists between the feeding arteries and draining veins of the brain linked by an interspersed abnormal intervening capillary bed - known as a central nidus [7-8]. Clinically, brain AVMs are a well-known cause of death, mostly due to the resulting intracerebral hemorrhage; however; they can remain asymptomatic for a very long time [8]. In the pediatric age, ruptured AVMs are considered a rare cause of sudden unexpected death. Cioca et al. report an exceedingly rare case of sudden non-traumatic death in a previously healthy nine-year-old boy due to a ruptured AVM of the choroid plexus of the fourth ventricle [7]. Another case by Racette et al. reported the sudden non-traumatic death of a 14-year-old girl who was previously well, except for mild asthma [9]. Examination of the brain revealed that a ruptured AVM caused an intracerebral hemorrhage [9]. In addition, Kerhunen et al. reported that brain AVMs were seen in 0.06% of medicolegal autopsies conducted in Helsinki (five out of 8038 consecutive autopsies) [10]. Clinically, only 12% of AVMs become symptomatic, with intracerebral hemorrhage being the most common clinical presentation [8]. Other presentations of AVMs include seizure, chronic headache, and focal deficits not related to hemorrhage [8]. It was reported that children who have brain AVMs have a worse prognosis than adults and a much higher bleeding tendency [8].

Although the majority of AVMs have a sporadic origin, familial cases have been reported in the literature [11]. Therefore, a histologic evaluation of grossly unidentified AVMs is of crucial value to surviving family members of the deceased [12]. It is noteworthy that very small malformations may not be evident on imaging or gross autopsy examination but rather found on histological examination. Therefore, it is recommended that forensic pathologists take numerous sections from hemorrhagic areas, including adjacent brain parenchyma and the choroid plexus [7,12].

Besides AVMs, other vascular malformations of concern include dural arteriovenous fistulas (DAVFs), cavernous malformations, and developmental venous anomalies (DVAs) [12]. DAVFs are vascular malformations that consist of abnormal connections between the arteries and veins of the dura mater [8]. They are responsible for approximately 10% to 15% of intracranial vascular malformations and unlike AVMs, they tend to be acquired and present later in life [12]. Cerebral cavernous malformations (cavernous angiomas) are clusters of irregular blood vessels lacking muscular or adventitial layers with no brain tissue present in between, whereas DVAs are persistent embryonic connections between the cortical and deep venous systems of the brain [8].

Tomcik et al. reported three cases of sudden nontraumatic intracerebral hemorrhage resulting from vascular malformations of the brain. In the first case, a 14-year-old girl presented with neck pain, headache, and vomiting of increasing severity [12]. On computed tomography (CT) scan of the brain, she was found to have an intraventricular hemorrhage with a midline shift [12]. Microscopic examination of the brain revealed a vascular malformation in the right thalamus and the cause of death was intraventricular hemorrhage [12]. Another case describes a 53-year-old man, a known hypertensive with elevated cholesterol, who was found dead at home by his wife [12]. Upon examination, it was found that he had a ruptured small cerebellar vascular malformation with associated intraparenchymal hemorrhage and subarachnoid hemorrhage [12]. Similarly, the cause of death of the last case reported was ruptured cerebellar vascular malformation. In all reported cases, the vascular malformations were not fully identified grossly but were rather confirmed by meticulous histologic examination. Hence, it is important for forensic pathologists to thoroughly investigate the underlying cause of hemorrhage by performing meticulous gross and histologic examinations for a more accurate assignment of the cause of death.

Cerebral Venous Thrombosis

Cerebral venous thrombosis is a rare cerebrovascular disease, occurring with an incidence of five per 1,000,000 per year [13]. It is defined as thrombosis of the intracranial veins and sinuses. Symptoms tend to be nonspecific such as headache, nausea, and vomiting, and are likely to be of longer duration rather than occurring suddenly. The most common but nonspecific symptom of sinus thrombosis is a headache, which mostly has an insidious onset and increases gradually over time. Etiologies behind CVT are numerous, including pregnancy, use of contraceptives, hypercoagulable states, and inflammatory and infectious conditions among others. CVT is often diagnosed late due to its highly variable clinical presentation, and this is mostly true in patients without known risk factors. CVT has a mortality rate ranging from 30% to 50%; however, current improved neuroimaging techniques have allowed improved diagnosis of pathologies that lead to precise intervention to achieve a good prognosis [8,13]. Non-contrast CT performed in an emergency setting may suggest the diagnosis of CVT, but magnetic resonance angiography (MRA) or digital angiography are the most effective techniques to diagnose the condition [8,13]. De-Giorgio et al. reported sudden death in a 31-year-old woman, a nonsmoker, who was not known to have any medical illness and was not using any contraceptives [13]. The patient initially presented with a history of intermittent headaches for two months at the nape of the neck with cervical pain not responding to nonsteroidal anti-inflammatory drugs (NSAIDs). Subsequently, she presented in an acute-onset comatose state with anisocoria. Head CT scan suggested the diagnosis of CVT but neither CT angiography (CTA) nor MRA was performed due to her hemodynamic instability. ﻿An autopsy revealed CVT that extended from the confluence of the sinuses to the right transverse sinus and tip of the superior sagittal sinus, in addition to a thrombus that obstructed the right internal jugular vein [13]. Screening for other causes such as toxicological or inherited prothrombotic factors was negative. ﻿As a result, spontaneous primary jugular vein thrombosis was the most likely cause of CVT [13]. One should have a high index of suspicion for CVT especially in young patients who develop an unusual headache or present with stroke-like symptoms in the absence of vascular risk factors and in patients with CT evidence of hemorrhagic infarcts [13].

Aneurysmal Subarachnoid Hemorrhage 

Subarachnoid hemorrhage (SAH) is a life-threatening emergency resulting from the extravasation of blood into the subarachnoid space. The leading cause of nontraumatic SAH is the rupture of an intracranial aneurysm, accounting for 80% of cases with high morbidity and mortality rates [14]. An intracranial aneurysm is defined as pathological dilatation of a localized part of an artery in the brain. Most aneurysms are true aneurysms located in the Circle of Willis and usually arise as solitary lesions [8]. Risk factors associated with intracranial aneurysmal hemorrhage include cigarette smoking, high blood pressure, heavy alcohol consumption, and the use of anticoagulants or contraceptive medications [8]. Lindbohm et al. reported that normotensive patients aged less than 50 years who never smoked and have an unruptured intracranial aneurysm are at low risk not only for rupture but also for sudden death from SAH [15]. Furthermore, Huang et al. estimated the overall probability of sudden death from aneurysmal SAH to be 12.4% with posterior circulation aneurysms contributing to 44.7% risk as compared to 12.1% for anterior circulation ones [16]. Moreover, Schievink et al. reported that 27% of patients suffering from ruptured posterior circulation aneurysms never reached the hospital as opposed to 9% of those with anterior aneurysms [17]. According to Freytag, 79% of subjects with basilar artery aneurysms died instantaneously compared to 59% of those with anterior circulation aneurysms [18].

A prospective study examining nontraumatic SAH found that 54% of patients suffered from a ruptured intracranial aneurysm causing sudden death; 14% of those died before receiving any medical attention while 40% died in the first 24 hours after hospitalization [14]. Additionally, Schievink et al. reported a similar percentage, which signifies the importance of early detection of aneurysms in preventing the possible risk of rupture [17].

Clinically, patients with aneurysmal SAH present most frequently with a sudden severe headache followed by loss of consciousness, nausea, vomiting, and focal neurologic deficits [8,14]. Complications of SAH include rebleeding and cerebral vasospasm. Rebleeding refers to a second rupture of the intracranial aneurysm and occurs in about 20% of patients after the initial bleeding and accounts for a 50% mortality rate [14]. Cerebral vasospasm is defined as narrowing of the arteries in the brain due to vascular spasm, which if severe enough can lead to stroke or death. Seven out of 10 patients with aneurysmal SAH develop vasospasm [14]. Rarely, in 10% to 15% of patients with SAH, no aneurysm or AVM is detected by angiography [19]. In these cases, the source of the bleeding is originating from perimesencephalic veins or capillaries [19]. These cases have a better prognosis than aneurysmal SAH and are less likely to rebleed [19].

Doberentz et al. reported a case of the sudden death of a 25-year-old woman who was diagnosed to have Alagille syndrome (AGS) [20]. The cause of death was SAH with invasion to the ventricular system resulting from a ruptured large saccular aneurysm of the terminal basilar artery. In patients with AGS, intracranial hemorrhage was reported in 14% to 16% of cases [20]. Vascular screening should be carried out in cases diagnosed with AGS as they have been reported in the literature to cause sudden unexpected death [20].

Cerebral Arterial Dissection

Cerebral arterial dissection (CAD) is one of the causes of stroke. Stroke has been reported as a cause of sudden death in 3% of cases, constituting the fourth most common cause of sudden death after sudden cardiac death, sudden death due to pulmonary embolism, and infectious diseases [21]. In 38% of these cases, no previous medical history was known [21]. When comparing hemorrhagic with ischemic stroke, hemorrhagic stroke is by far the most frequently reported cause of death in stroke cases, contributing to 94% of sudden death incidents compared to 6% caused by ischemic stroke [21-22]. Clinically, 19% of patients present with neurological symptoms one to 24 hours prior to their death, with headache being the most common complaint [21]. Other symptoms include decreased level of consciousness and weakness or numbness of the extremities [21].

Matschke reports the interesting finding of CAD in four cases as a cause of sudden death due to ischemic stroke in children [23]. Risk factors for ischemic stroke in children include congenital heart disease, sickle cell anemia, infectious and inflammatory disorders, and primary vasculopathies [23-25]. Yet, in several patients, the cause remains undetermined [23-24]. CAD has been increasingly identified in current literature as a cause of stroke [23-28]. It was found that there is a male predominance among children with CAD and unlike in adults, CAD in pediatric age tends to occur most frequently in intracranial vessels [27]. In cases reported by Matschke, cerebral infarctions were mainly found in the territory of the middle cerebral artery (MCA) due to dissection of the left MCA [23]. Childhood CAD carries a poor prognosis with a 33% mortality rate in anterior circulation arterial dissections and 2% in posterior ones [29].

*Epilepsy* 

Sudden unexpected death in epilepsy (SUDEP) is defined as sudden, unexpected, nontraumatic, non-drowning, witnessed or unwitnessed death occurring in an otherwise healthy individual with epilepsy with or without evidence of seizure in which postmortem examination is unrevealing [30-32]. SUDEP is classified into definite, probable, and possible. In definite SUDEP, an autopsy has confirmed the absence of anatomical or toxicological cause of death [30-31]. In probable SUDEP, an autopsy has not been done but the circumstances of death are strongly suggestive of SUDEP while possible SUDEP describes a situation in which SUDEP cannot be excluded and should be considered among the explanations of death [30-32]. Patients diagnosed with epilepsy have a nearly 24 times higher risk of sudden unexpected death compared to the general population [33]. Moreover, patients with chronic refractory epilepsy were shown to have a higher risk of SUDEP [32].

Despite the extensive literature on SUDEP, the pathophysiology underlying SUDEP remains uncertain, and it is likely due to more than only one mechanism. Peri-ictal impairment of respiratory function, post-ictal impairment of brain function, cardiac arrhythmias, and autonomic dysfunction have all been implicated as mechanisms [34-36]. Most cases of SUDEP are unwitnessed and are infrequently documented, which makes it difficult to ascertain the mechanism of sudden death. In addition, very few studies include ECG, EEG, or oxygen recordings during sudden or near-sudden death in epilepsy [36]. However, certain risk factors have been discussed in the literature that predispose patients with epilepsy to sudden death. An important risk factor is the presence of generalized tonic-clonic seizures (GTCS). Langan et al. compared the autopsies of 154 SUDEP cases with matched controls and found that the risk of SUDEP is 14 times higher when a history of GTCS was reported in the previous three months [37]. Hesdorffer et al. report that compared to no GTCS episodes, having three or more GTCS episodes in the past year carries a 10-fold increased risk for SUDEP [31]. Other important risk factors for SUDEP include nocturnal seizures, increased seizure frequency, cognitive delay, young age at epilepsy onset, longer duration of epilepsy, and dementia [31,34]. Studies have been inconsistent on the potential effect of antiepileptic drugs (AEDs) on SUDEP; however, a recent review article concluded that AEDs are not associated with increased risk for SUDEP [31]. Additionally, a recent meta-analysis of randomized controlled trials provided strong evidence that treatment with AEDs increased seizure control and played a role in the prevention of SUDEP [38].

Sirbu reported a rare finding of dysembryoplastic neuroepithelial tumor (DNT) in a 25-year-old woman who was known to have refractory epilepsy [39]. DNT is a low-grade mixed neuronal and glial tumor composed of oligodendrocytes, astrocytes, neurons, and glyconeural elements [39]. The most frequent presentation of this tumor is partial seizure followed by signs and symptoms of increased intracranial pressure. In this case, secondary generalization of seizures caused by the diffuse left temporoparietal DNT has led to the probable SUDEP [39].

Heterotopias were reported in the literature to be a cause of SUDEP. These are lesions formed of aggregates of neuronal and glial cells resulting from impaired neuronal migration allowing multiplication of neuroblasts but preventing their migration from the periventricular region to the cerebral cortex [40-41]. In a case reported by Matschke et al., a 35-year-old man who was in good health apart from being a heavy smoker was found lying on the floor unconscious [40]. Autopsy revealed acute infection of the upper airways with edema and congestion of the bronchial mucosa in addition to fresh bite marks on the tongue [40]. Neuropathologic examination showed bilateral nodules of gray matter around the occipital horns of the lateral ventricles bulging into the ventricular lumen suggesting a subependymal nodular heterotopia [40]. These lesions had infiltrates of T lymphocytes intermingled with some B lymphocytes on immunohistochemistry [40]. In addition, further immunohistochemical studies failed to identify multiple viral antigens [40]. In this case, the patient had subependymal nodular heterotopia and benign lymphocytic (aseptic) meningitis resulting in an epileptic seizure as the cause of sudden death [40].

Neuroimaging is a very useful tool in discovering structural and functional brain abnormalities that could identify the underlying mechanism of death and provide markers to identify high-risk patients [42]. For instance, magnetic resonance imaging (MRI) can demonstrate structural brain tissue abnormalities and disturbance of functional connectivity, particularly within cortical and subcortical areas. Hence, it would enable the identification of high-risk patients who are predisposed to SUDEP [42].

Primary Brain Tumors

The incidence rate of CNS tumors worldwide is approximately 4.63 per 100,000 persons [43]. Globally, in 2016, there were 227,000 documented deaths with a death rate accounting for 3.24 per 100,000 persons [43]. Additionally, in a medicolegal autopsy series, sudden unexpected death due to primary undiagnosed CNS tumors accounted for 0.02% to 2.1% of cases [44]. The most-reported histological types are neuroepithelial tissue tumors, which contribute to 28.2% of all primary CNS tumors [45-46]. These include astrocytic, oligodendroglial, ependymal, and embryonal tumors among others. Among these, astrocytic tumors are considered the most common type of brain tumor, accounting for more than 38% of all primary CNS lesions [47]. A review of cases of sudden unexpected death based on the World Health Organization (WHO) classification system of CNS tumors is provided. A total of 21 cases diagnosed with astrocytic tumors was reported by Opeskin et al. [47]. In one of the cases, a 20-year-old woman suffering from neurological symptoms since the age of 11 months was found unconscious at home [47]. The autopsy revealed a diffuse low-grade astrocytoma (WHO grade II) located in the brainstem, involving the reticular formation, nucleus ambiguus, and solitary tract [47]. The autopsy on another two cases revealed anaplastic astrocytoma (WHO grade III), which presented with neurological symptoms before the time of death. Another case describes a 67-year-old man who presented to the hospital with a diagnosed brain tumor a month prior to his death [45,48]. Similarly, a 36-year-old man presented to the hospital with a headache and other neurological symptoms prior to death [45,48]. Other astrocytic tumors reported to cause sudden death include two cases of subependymal giant cell astrocytoma and another two with pilocytic astrocytoma [49-51]. Moreover, 12% of all cases of sudden unexpected death due to primary intracranial tumors are due to glioblastoma multiforme (GBM) (WHO grade IV). Riezzo et al. reported a total of 13 cases of sudden death due to undiagnosed GBM, of which eight had no prior neurological symptoms while the remaining four had symptoms like headaches and non-specific cognitive or personality changes [44]. The last case was a case of undiagnosed GBM found in a 10-week-old infant who presented with irritability and vomiting prior to death [44]. In a case series including 11 cases of undiagnosed primary CNS tumors, seven glial tumors were reported: one diffuse astrocytoma (grade II), one anaplastic astrocytoma (grade III), one malignant glioma not otherwise specified, and four GBM (grade IV) [45]. The remaining four cases included one schwannoma, one pituitary adenoma, and two colloid cysts [45]. The most frequently reported mechanism of death among the cases was increased intracranial pressure.

Meningiomas are mostly benign meningothelial cell neoplasms, typically originating from the inner surface of the dura mater and represent 37.1% of all intracranial tumors [46]. Gitto et al. reported a total of 12 cases with sudden unexpected death occurring due to unrecognized intracranial meningioma [52]. Postmortem examination reports different etiologies that demonstrate various mechanisms of death in cases of meningiomas [52]. For instance, peritumoral brain edema, which causes increased intracranial pressure, was found to be the mechanism of death in a total of five cases [52]. However, three of these cases showed complications of direct compression to the adjacent structures most commonly the cerebellum and the brainstem, which are crucial for monitoring metabolic processes and regulating other autonomic nervous system activities [52].

Pituitary adenomas are the most frequently reported CNS neoplasms, accounting for 16.8% of all intracranial tumors [46]. They are classified into micro and macroadenomas. Two fatal cases reported by Salami et al. presented with altered mental status and neurological symptoms in addition to a history of visual loss [53]. Postmortem examination of both cases revealed giant pituitary adenomas [53]. Among the important complications of pituitary adenomas is pituitary apoplexy, which occurs either because of pituitary tumor bleeding or compromise of blood supply causing necrosis and swelling [54]. Two cases reported sudden death due to undiagnosed pituitary adenomas complicated by pituitary apoplexy [53-54]. In both cases, the postmortem examination revealed a mass with extension to suprasellar regions and adhesions to the adjacent structures [53-54].

Embryonal tumors are a group of CNS tumors that are highly malignant and classified histologically as neuroepithelial in origin. Among the common types of embryonal tumors is medulloblastoma, which is a highly malignant tumor that is seen more commonly in children. Although adult cases of medulloblastoma account for less than 1% of all adult intracranial tumors, two cases reported sudden death resulting from adult-type medulloblastoma [55-56]. The first case was a 21-year-old man who was not known to have any medical illness while the second case was a 28-year-old pregnant woman at the 33-week-gestational period [55-56]. In children, the incidence rate of medulloblastoma peaks at the age of nine years and younger [46]. Four cases have been reported in the literature where the cause of sudden death was documented to be undiagnosed medulloblastoma [46,57].

Schwannomas are non-malignant nerve sheath tumors that are usually located in the cerebellopontine angle, which account for 10% of all benign intracranial tumors [58]. A review of the literature showed two cases of pregnant women who presented with headache and vomiting and had to be admitted to the intensive care unit (ICU) but deteriorated to death shortly after [45,58]. The cause of death by postmortem examination was found to be schwannomas [45,58].

Colloid cysts are benign neuroepithelial tumors accounting for less than 2% of all intracranial masses and represent 0.001% to 0.009% of cases of sudden unexpected death [59]. A systematic review by Lagman et al. identified a total of 109 patients with colloid cysts [59]. Eighty-seven of these cases were seen in adults while 18 cases were seen in children [59]. The most common signs and symptoms reported prior to death were headache, nausea, vomiting, loss of consciousness, and papilledema. The most frequent mechanism of death was acute obstructive hydrocephalus [59-61].

Gangliocytomas and gangliogliomas are low-grade neuroepithelial tumors in origin that represent 1.3% of all CNS tumors [62]. Takahashi et al. reported a case of a 30-year-old man who was known to have cerebral palsy with a long history of gait disturbance, repeated falling, and strabismus, and had collapsed suddenly in bed [62]. The autopsy revealed the finding of gangliocytoma located in the medulla oblongata [62]. Moreover, gangliogliomas are neoplastic tumors with a glial component reported to be the cause of sudden death in several cases in the literature and were found most frequently located in the brainstem [63].

Oligodendrogliomas are relatively uncommon CNS tumors that arise from the oligodendrocytes. These tumors are rarely reported to cause sudden neurologic death. Manousaki et al. described a case of a 28-year-old man who was found dead in his apartment [64]. Autopsy of the case concluded the diagnosis of a hemorrhagic well-differentiated oligodendroglioma [64].

Choroid plexus xanthogranulomas are benign CNS masses that are most frequently located in the choroid plexus of the lateral ventricle and account for 1.6% to 7% of cases [65]. In a 51-year-old man who was found dead, postmortem examination revealed the cause of death to be a choroid plexus xanthogranuloma with resultant intraventricular and subarachnoid hemorrhage [65].

Other primary intracranial tumors reported in the literature to cause sudden unexpected death are exceedingly rare. They include central neurocytomas, intracranial hemangiopericytomas, meningeal melanocytosis, intracranial teratoma, and calcifying pseudoneoplasm of the neuraxis (CAPNON).

Metastatic Brain Tumors

Brain metastasis is reported in 20% to 25% of all cancer patients and autopsy findings report intracranial metastasis to be the cause of death in up to 25% of cases [66]. Lee et al. described a case of a 22-year-old man who shortly prior to death experienced episodic headaches and later was found lying unconscious by his friends [67]. Postmortem CT scan showed a prevertebral retroperitoneal mass, a lesion in the right frontal lobe compressing the right lateral ventricle, and distinct masses in the lung, liver, and kidney [67]. Internal examination revealed a retroperitoneal mass that was diagnosed histologically as a malignant mixed germ cell tumor with metastatic nodules to the brain, lung, liver, and left kidney [67]. The cause of death was reported to be increased intracranial pressure due to brain metastasis occurring as a result of metastatic retroperitoneal malignant tumor [67].

Malignant melanoma is a neoplasm derived from melanocytes. In fact, it is the third most common cause of CNS metastasis and the five-year cumulative risk of malignant melanoma metastasizing to the CNS was reported to be 7% [66]. Postmortem examination in a 48-year-old man who was found unconscious on the bedroom floor showed a neoplastic mass with cerebral and cerebellar hemorrhages along with multiple cutaneous nevi [66]. Histological and immunohistochemistry analysis confirmed the cause of death to be metastatic melanoma [66].

Meningitis

Sudden death due to meningitis is rarely reported in the literature. A recent article describes the time and cause of death in patients with community-acquired bacterial meningitis above the age of 15 years [68]. In a retrospective study, six (7%) out of 84 patients had sudden unexpected death [68]. Moreover, out of 18 patients suffering from CNS complications, eight were reported dead in less than 48 hours [68].

Cerebral Abscess

Brain abscesses account for approximately 8% of intracranial masses in developing countries [69]. A ruptured brain abscess into the subarachnoid space was responsible for the sudden death of a middle-aged man [69]. Two weeks prior to his death, the patient had a history of fever, purulent rhinorrhea, and headache, indicating that the most likely source of abscess spread was a sinus infection [69]. The autopsy confirmed the presence of two frontal abscesses as well as abscess exudate found in the left frontal and left occipital lobes with resultant meningeal inflammation [69]. Microscopically, there were gram-negative rods and gram-positive cocci clusters [69]. Furthermore, the patient had a history of an atrial septal defect, which might have predisposed him to paradoxical embolisms and cerebral abscesses [69].

The role of neuroimaging and genetic testing as autopsy ancillary investigations in CNS-related sudden death

In addition to history and examination, neuroimaging and a molecular autopsy could be beneficial for the forensic pathologist to conclude the cause of death in cases of sudden death due to CNS causes. For instance, the most important and sensitive sequences in postmortem brain MRI used in the evaluation of intracranial hemorrhage are gradient recall echo (GRE) and susceptibility-weighted imaging (SWI) [70]. On GRE, hypointensity signifies hemoglobin byproducts and can be appreciated in an acute parenchymal hematoma [70]. Similarly, hypointensity on SWI characterizes hemorrhage [70]. Careful evaluation of these sequences will help differentiate hemorrhage from other causes of hyperattenuation on CT such as calcifications or highly vascular tumors. In a retrospective study involving 57 cadavers conducted to correlate postmortem forensic neuroimaging with autopsy results, MRI and CT imaging showed a sensitivity of 63% in the detection of hemorrhages in the white matter [71]. Moreover, postmortem MRI and postmortem CT were superior to the autopsy in the evaluation of intraventricular hemorrhage [71]. Additionally, regarding the cause of death, brain CT and MRI showed an overall good correlation with autopsy [71].

A conclusive cause of death could be ascertained in up to 65% of cases after macroscopic evaluation (positive macroscopic autopsy) [72]. However, for the remaining cases in which an inconclusive autopsy (negative macroscopic autopsy) is reported, molecular autopsy (postmortem genetic testing) plays a major role. A molecular autopsy has the potential to improve the diagnosis of sudden unexpected death, which is especially crucial to surviving family members. One of the most cost-effective techniques in molecular autopsy is next-generation sequencing (NGS) particularly whole-exome sequencing (WES). WES has the ability to rapidly analyze the entire coding sequence of the human genome, identifying rare, previously unknown variants. In one study involving 25 victims of sudden unexpected death, 18 were found to have mutations potentially causing sudden unexpected death [73]. One drawback of WES is that a great number of variants must be assessed to identify the potentially causal variant. For this reason, the human phenotype ontology (HPO) annotation is used internationally for phenotyping rare diseases [74]. A study used, for the first time, an HPO-based evaluation of WES in sudden unexpected death [74]. It discovered 13 potentially causative variants but only one was classified as pathologic, which is the homozygous variant in UPB1. This variant has been recently known to trigger seizures due to b-ureidopropionase deficiency in a recessive mode of inheritance [74]. Similarly, Assmann et al. reported the same variant in a four-month-old infant with an acute life-threatening event with febrile status epilepticus [75]. Furthermore, whether a patient develops neurological symptoms as well as severity is largely determined by the extent of reduction in enzyme activity caused by the variant along with other environmental factors [74]. Consequently, in many affected individuals with absent or mild neurological problems, the condition may never be diagnosed [74]. Additionally, postmortem molecular autopsy of SUDEP cases discovered variants in genes encoding for sodium and potassium ion channel subunits, and 18 genes and four different duplications have been reported to have a possible link to SUDEP [76].

## Conclusions

Sudden unexpected death is one of the important issues in forensic pathology. Sudden death due to CNS-related pathologies is less common when compared to cardiovascular causes. The CNS-related pathologies causing sudden death commonly encountered in forensic autopsy practice include vascular malformations, aneurysmal subarachnoid hemorrhage, epilepsy, brain tumors, and infectious causes like meningitis and cerebral abscess. Neuroimaging and genetic testing are important autopsy ancillary investigations that help in the diagnosis of such cases. Besides, a molecular autopsy in such cases helps in the identification of relatives of the deceased who are at high risk of being predisposed to sudden death from a similar cause, for instance, SUDEP.
